# Usability of iSupport Swiss, a World Health Organization Digital Intervention for Caregivers of People With Dementia: Mixed Methods Study

**DOI:** 10.2196/81247

**Published:** 2026-04-15

**Authors:** Anna Messina, Rebecca Amati, Anna Maria Annoni, Emiliano Albanese, Maddalena Fiordelli

**Affiliations:** 1 Institute of Public Health, Faculty of Biomedical Sciences, Università della Svizzera italiana, Via Buffi 13, Lugano, 6900, Switzerland, 41 0782104055

**Keywords:** eHealth, dementia, caregiver, psychosocial interventions, usability

## Abstract

**Background:**

Dementia presents a pressing public health challenge, with informal caregivers (ICs) playing a pivotal role in supporting people with dementia. Digital interventions, such as the World Health Organization (WHO) iSupport, offer scalable, self-guided psychosocial and educational support for caregivers. However, effective implementation relies on strong usability, particularly for older adults with varying levels of digital literacy.

**Objective:**

This study aimed to evaluate the usability of the desktop version of iSupport Swiss, a WHO culturally adapted digital intervention for ICs of people with dementia in the Italian-speaking region of Switzerland.

**Methods:**

We conducted a mixed methods usability study at the Università della Svizzera italiana. Participants (formal caregivers and ICs) completed a pretest questionnaire to collect sociodemographic data and digital health literacy measures via the Italian version of the eHealth Literacy Scale, 11 structured usability tasks, a posttest System Usability Scale, and a semistructured interview. Task performance was observed and analyzed, including completion rates and qualitative feedback obtained through the think-aloud technique. Interviews were analyzed using thematic analysis.

**Results:**

Twelve caregivers (10 ICs and 2 formal caregivers) took part in the study. Most participants showed high digital proficiency (Italian version of the eHealth Literacy Scale: mean 31.17, SD 5.84) and completed most tasks successfully or with minimal support. The mean System Usability Scale score was 71.5 (SD 12.27), indicating good usability. Website features were rated positively, especially information quality (mean 4.58/5, SD 0.51) and quantity (mean 4.42/5, SD 0.51), though ease of navigation scored lower (mean 3.42/5, SD 1.00). Qualitative interviews revealed 5 core dimensions: content quality and relevance, credibility of the source, navigation and usability, interactivity, and emotional impact. Suggestions included clearer language switching, improved navigation cues, and more personalized feedback in exercises.

**Conclusions:**

iSupport Swiss demonstrated overall good usability among caregivers, supporting its further implementation. Key areas for refinement include navigation clarity and user-tailored feedback. Participants appreciated the emotional resonance and credibility of the program, underlining its potential to enhance caregiver self-efficacy and early help-seeking. Usability testing proved essential in identifying both functional and affective dimensions of user experience. Future work should extend to broader and more diverse populations, incorporate real-world usage settings, and examine long-term engagement to optimize adoption and impact. Successful use and further development of iSupport, beyond the specific Swiss context, could contribute to expanding the availability of accessible and appropriate resources for ICs, ultimately helping to reduce caregiver burden and alleviate its impact on social and health care systems globally.

## Introduction

### Background

Dementia poses a significant and growing public health challenge globally [1], and Switzerland is no exception. The burden of dementia is substantial and complex, with marked implications at the family and societal levels. With over 150,000 individuals currently living with dementia [2] and projections estimating a doubling of this number by 2050, the need for effective support systems is urgent, particularly for family caregivers, also known as “informal caregivers” (ICs), who serve as the primary source of care for people living with dementia [3].

Over the past 2 decades, digital health (eHealth) interventions have emerged as scalable and accessible means to provide psychosocial and educational support to ICs [4,5]. These solutions are considered particularly well-suited for caregivers, offering flexibility in terms of time and location, which is critical given the time and organizational constraints of dementia care and its complexity [6]. Despite the potential of eHealth interventions to alleviate the burden and improve the quality of life for both caregivers and people with dementia, several challenges may hamper their successful implementation and uptake. A scoping review of 32 studies reported an average retention rate of approximately 70%, indicating that nearly one-third of participants discontinued their involvement [7]. More recently, a systematic review including 18 studies involving more than 2000 caregivers found an overall pooled dropout rate of 18.4% [8]. In the context of the World Health Organization (WHO) iSupport adaptation in Spain [9], the authors highlighted the risk that eHealth interventions may primarily reach a narrow segment of digitally engaged users rather than the broader population of caregivers for whom they are intended. Overall, evidence on actual adoption and reach is extremely scarce, and properly designed implementation studies that test the combined effectiveness, scalability, and sustainability of eHealth interventions for caregivers are still lacking. Existing literature provides more consistent insights into the factors associated with attrition during experimental and feasibility stages. These factors can be broadly categorized into content-related challenges (eg, a gap between caregivers’ needs and the solution or competing care priorities) [8] or technology-related challenges (eg, interface design, digital readiness, and ease of navigation) [10,11]. The latter may be particularly pronounced among older caregivers, who may have relatively low levels of digital literacy and technological proficiency [12].

Usability studies play a crucial role in ensuring that the interactive system is tailored to users’ needs and skills and play a key role in informing implementation. According to Bastien [13], usability assessment focuses on 3 main dimensions: effectiveness, the degree to which the system achieves its intended functions; efficiency, the resources, such as time and effort, required to accomplish specific tasks; and user satisfaction, the extent to which users experience positive attitudes and responses while using the system. Additionally, usability studies are instrumental in uncovering design problems and errors, as well as in suggesting potential refinements before an effectiveness evaluation [14].

This study aims to evaluate the usability of the desktop version of iSupport Swiss [15], which is presented in detail in the “Overview of iSupport Swiss” section.

### Overview of iSupport Swiss

iSupport Swiss is a culturally and linguistically adapted version of the original iSupport program developed by the WHO to provide support and education to ICs of people living with dementia [16], tailored to the Swiss context and culture [15]. The adaptation process was based on a community-based participatory approach [17], incorporating feedback from family caregivers, health care professionals, and relevant stakeholders. The active engagement of all stakeholders enabled the modification of the original content to better reflect the linguistic preferences, cultural values and preferences, informational needs, and caregiving practices specific to the Swiss population. Accordingly, adaptations were made across various domains, including language, resources, content, visual design, and functional features, to ensure cultural relevance. A detailed description of the adaptation procedures and the specific modifications implemented can be found in prior publications [15,18].

Since 2023, iSupport Swiss has been available in both online and print formats. The digital version is freely accessible as a mobile app (for smartphones and tablets) and as a desktop website. Consistent with the original WHO iSupport tool, the program consists of 22 chapters organized into 5 core modules, each addressing key aspects of caregiving. These include both practical support for care recipients (eg, assistance with toileting, personal hygiene, and nutrition) and strategies for caregiver well-being (eg, stress management and mobilizing support networks). Each chapter combines theoretical background with practical guidance, illustrated through real-life case scenarios and interactive components, such as multiple-choice and open-ended questions. Upon accessing the homepage, users are prompted to register or log in using an email address and password. Following authentication, users are asked to complete 2 brief multiple-choice questions regarding their caregiving situation and their reasons for accessing the program. Based on their responses, a personalized selection of chapters is suggested. By default, users are encouraged to begin with the introductory guide (chapter 0). They can then either proceed with the recommended content or freely browse the full list of available chapters. Screenshots from the program are available in Multimedia Appendix 1.

## Methods

### Ethical Considerations

Prior to starting the study, the project (2020‐02030/CE 3731) was submitted to the Cantonal Ethics Committee, which reviewed and evaluated the study protocol. Following their assessment, the committee concluded that the project does not qualify as research involving human participants under Article 3 of the Swiss Human Research Act (Loi relative à la recherche sur l’être humain) [19], as it did not involve the collection of sensitive health-related data or clinical interventions. The committee also confirmed that no ethical concerns were identified in relation to the study procedures. All participants received written information about the study, were informed about confidentiality and data protection, and provided written informed consent prior to participation. No financial compensation or reimbursement was provided to participants for their involvement in the study.

### Study Procedure and Sample

The study was conducted in the Italian-speaking region of southern Switzerland, namely the Canton of Ticino. We conducted this study in accordance with the WHO iSupport adaptation guidelines [16], which also recommend locally testing the intervention before its final implementation. The procedures described in this study represent a direct continuation of the cultural adaptation process of iSupport in Switzerland, the methodology of which has already been detailed elsewhere [15].

Before conducting the usability study, we pretested the usability tasks with the members of the iSupport Community Advisory Board (CAB). Members of the CAB included caregivers of people with dementia, representatives of the project’s funding agencies, and other national collaborating partners who had participated in the previous cultural adaptation process of iSupport Swiss.

For this study, we recruited both formal caregivers (FCs) and ICs: the former as prospective target users of iSupport and the latter as potential facilitators and communicators of the program. In January 2022, we drafted an invitation letter and distributed it to the members of the CAB and their associations and institutions, as well as to participants from previous phases of iSupport adaptation or other ongoing projects who had consented to receive information about further research activities. Eligibility criteria for ICs included: (1) having (at present or in the past) experience in caring for a person with dementia, (2) being fluent in Italian, and (3) being familiar with digital technology. Inclusion criteria for FCs included: (1) having experience in dementia care, (2) being fluent in Italian, and (3) being familiar with technology. We sent the informed consent forms to participants who contacted us and met the inclusion criteria. Once we received the signed consent forms, we called them to arrange their sessions.

### Data Collection

All participants’ sessions were carried out in person in a laboratory setting at the Università della Svizzera italiana between January 28, 2022, and February 10, 2022. The sessions were conducted by 1 facilitator with expertise in iSupport (AM) and 1 observer experienced in qualitative research methods (IF). We provided participants with a personal desktop computer with internet access.

The usability sessions consisted of four distinct steps: (1) pretest questionnaire, (2) usability tasks, (3) posttest questionnaire, and (4) posttest interview. The methodology combined quantitative and qualitative approaches, implemented sequentially across the usability sessions.

Quantitative methods were used during the first 3 phases to collect sociodemographic information and to assess participants’ interaction with the system during task performance, including task completion, need for assistance, and usability ratings.

Qualitative methods were applied both during and after the usability tasks. During task performance, qualitative data were collected through the think-aloud technique and observational notes to capture immediate, in-task feedback (“real-time” reflections) on users’ interactions with the system.

Following task completion, qualitative methods were further applied in the final phase through 4 open-ended questions, allowing an in-depth exploration of participants’ subjective experiences, perceptions, and emotional responses.

During the pretest questionnaire, we asked participants to fill out an online questionnaire via REDCap (Research Electronic Data Capture; Vanderbilt University) [20]. Through this survey, we collected sociodemographic characteristics (age, sex, occupational status, and type of caregiver), caregiving data (living situation of people with dementia and the time spent caregiving), and measured the frequency of average internet use (more than once a day, at least once a week, or less than once a week), the type of devices used (smartphone, tablet, and computer), and health digital literacy. The latter was measured through the validated Italian version of the eHealth Literacy Scale (IT-eHEALS) [21], an 8-item standardized scale developed to measure users’ skills and knowledge in finding, evaluating, and using eHealth information to address health issues [22].

For the usability tasks, we based our procedures on the testing protocol of the iSupport-Portuguese version [23] and on previous studies and recommendations in co-designing health interventions [13,24,25].

As part of the usability test material, we provided participants with a hard copy guide that described 11 tasks (Multimedia Appendix 2) to be performed using the iSupport website. The tasks ranged from easier tasks (eg, entering the website and completing the login procedure) to more complex activities (eg, navigating a chapter and completing exercises).

Participants were instructed to try to perform the task autonomously, with the possibility to ask for support from the facilitator (AM), and to verbalize their navigation behavior by sharing impressions and comments, according to the “think-aloud technique” [26]. Assistance was not provided unless explicitly requested by the participant, in order to replicate behavior in a more realistic and naturalistic context. In parallel, the observer (IF) filled a structured observation sheet to collect the following data: task completion (categorized as concluded, partially concluded, concluded with support, or not concluded), task completion time (minutes taken to complete the task), main difficulties encountered, and user verbalization. We recorded participants’ navigation behavior and commentary using audio recorders and screen-capturing software (Panopto).

After completing the tasks, participants were asked to fill out a posttest questionnaire on REDCap. We measured usability with an Italian back-translated version of the System Usability Scale (SUS) [27], a widely used 10-item Likert scale that measures the subjective assessment of a system’s usability. The final SUS score ranges from 0 to 100, with higher values representing better usability, and a standard cutoff of 68 for defining a usability score as above or below average [27].

Based on literature recommendations [26,28], we also provided participants with a list of 34 adjectives that users could choose from to best describe the website, along with a rating scale (1-5) for website features (colors, font type and size, quantity and quality of information, ease of navigation and reading, and design and graphics). The choice of adjectives was informed by the User Experience Questionnaire by Schrepp [29].

The final phase included 4 open-ended questions aimed at gathering participants’ perspectives on the usability and usefulness of the website. More specifically, participants were asked what they had liked and what they had not liked about the platform, whether they would use it and why, and if they would recommend it to others. They were also asked how using the platform had made them feel and whether they had any additional comments. Given the targeted scope of the interview and the limited sample, interviews were conducted with all participants included in the study.

During the tasks, the observer asked follow-up questions to further investigate and clarify specific observations noted throughout the sessions. This final qualitative phase allowed us to complement and deepen the interpretation of the usability findings, providing insights into observed task performance patterns and usability scores within participants’ subjective perspectives.

All interviews were audio recorded, transcribed verbatim, and analyzed using an inductive thematic analysis approach [30].

Following transcription, 2 researchers (AM and AMA) independently familiarized themselves with the data, generated initial codes, and iteratively grouped them into candidate themes. Themes were then reviewed, refined, and agreed upon through discussion to reach consensus. A high degree of convergence in participants’ responses was observed across interviews, suggesting that the collected qualitative data were sufficient to inform the usability evaluation and complement quantitative findings. Integration of qualitative and quantitative data occurred during the interpretation phase, by comparing task performance metrics, usability scores, and website ratings with qualitative findings from think-aloud comments and interviews.

## Results

### Characteristics of Participants and Context of Care

Twelve participants were involved in the study: 8 women and 4 men, with a mean age of 56.5 (SD 9.64) years. Most participants (n=10) were ICs, providing care for a family member with dementia. Two participants were FCs, more specifically home nurses. Seven cared for an older adult living independently, while in the other cases, the person with dementia lived in the caregiver’s home (n=2) or was in a care facility (n=1). Seven participants had between 1 and 2 years of caregiving experience, while 2 had over 10 years of experience. Most caregivers accessed the internet more than once a day (n=10), using either a computer (n=6), a tablet (n=4), or a smartphone (n=2).

The mean score on the IT-eHEALS was 31.17 (SD 5.84; range 23‐40), indicating a high level of proficiency in locating, evaluating, and using electronic health information for decision-making. Table 1 summarizes the main characteristics of participants, including scores from the IT-eHEALS.

**Table 1.** Characteristics of formal and informal caregivers of people with dementia in the usability study (N=12).

Characteristics	Value	
Age (y), mean (SD)	56.5 (9.9)	
Female, n (%)	8 (66.7)	
Occupational status, n (%)		
Employed	8 (66.7)	
Housewives	2 (16.7)	
Retired	1 (8.3)	
Missing	1 (8.3)	
Caregiver, n (%)		
Formal	2 (16.7)	
Informal	10 (83.3)	
People with dementia alive, n (%)	7 (58.3)	
People with dementia living situation, n (%)		
Community-dwelling	7 (58.3)	
Caregiver’s home	2 (16.7)	
Nursing home	1 (8.3)	
Missing	2 (16.7)	
Caregiving experience (y), n (%)		
1‐2	7 (58.3)	
3‐5	2 (16.7)	
6‐10	1 (8.3)	
>10	2 (16.7)	
Internet use, n (%)		
More than once a day	10 (83.3)	
At least once a week	2 (16.7)	
Device, n (%)		
Smartphone	2 (16.7)	
Tablet	4 (33.3)	
Computer	6 (50)	
IT-eHEALS^a^, mean (SD)	31.17 (5.84)	

a IT-eHEALS: Italian version of the eHealth Literacy Scale.

### Task Performance

Both FCs and ICs successfully or partially completed most tasks, with a success rate exceeding 70%. Participants who required assistance to complete a task were classified as having partially completed the task or completed it with support. Partial or no success was mainly related to task 2 (changing language options after the login procedure), task 5 (going to the homepage using a shortcut), task 6 (distinguishing the difference between the modules and chapters and clicking on the respective links), and task 10 (returning to the previous chapter using a shortcut). Task performance is summarized in Table 2.

**Table 2.** Task performance of formal and informal caregivers using the iSupport Swiss website.

Task	Completed, n (%)	Partially completed, n (%)	Noncompleted, n (%)	Task completion time in minutes	Type of errors
1. Go to the login page	12 (100)	—^a^	—	<1	N/A^b^
2. Login procedure and change language	9 (75)	3 (25)	—	2	Unable to find the language settings
3. Fill the navigation survey	11 (92)	1 (8)	—	2	Unable to provide only one answer Difficulty to see the button “send the survey”
4. Browse the list of suggested chapters	12 (100)	—	—	6	N/A
5. Going to the homepage	7 (58)	4 (33)	1 (8)	4	Unable to go take the shortcut from the opened chapter to the homepage
6. Select a module and a course	4 (33)	4 (33)	4 (33)	6	Unable to understand the difference between modules and chapters and to click on the respective links
7. Open and read a chapter	10 (83)	2 (17)	—	6	N/A
8. Change chapter	8 (67)	3 (25)	3 (25)	1	N/A
9. Fill an exercise or activity	11 (92)	1 (8)	—	9	Unclear whether the answers are read by someone or not Exercises are funny and useful as positive reinforcement The chance to have feedback on the exercises is appreciated but not so visible
10. Return to the previous chapter	6 (50)	6 (50)	—	7	Unable to take the shortcut
11. Logout procedure	8 (67)	4 (33)	—	<1	N/A

a Not applicable.

b Not available.

The think-aloud procedure allowed us to collect impressions and real-time feedback during the task performances. For instance, 1 participant commented on their first impression while landing on the login page (task 1): “Yellow gives you the strength to act; the color is balanced” (ID4, IC).

On the same task, 2 participants suggested adding a sentence detailing the topic of the program:

*The colors are neutral; a welcome message is important*

[ID5, FC]

*There is no initial explanation of what it is, who it is for, I suggest putting in large font “for family caregivers….”*

[ID2, IC]

On task 2, few participants took the time to change languages:

*I expected to find macro themes on this page,…the language icon should be standardized.*

[ID3, IC]

*I don’t understand how to proceed, it is not clear how to change the language*.

[ID6, IC]

Task 3 required participants to answer 2 multiple closed-ended questions to suggest a thematic path. While some participants found them useful for focusing on their situation, others found them not enough and expected more:

*The questions are helpful, they help me understand where I am.*

[ID7, IC]

*The second question already requires a level of self-reflection that caregivers might not have, I was expecting more guiding questions....*

[ID9, FC]

Task 4 required participants to browse the list of suggested chapters based on the answers provided during the previous task. While first impressions were generally positive, some participants did not realize that this was a suggested thematic path and not a comprehensive list of all chapters. Comments included, “Great, let’s get straight to the point!” (ID4, IC) and “It’s not very clear that this is a selection” (ID12, IC).

Consequently, some caregivers became confused when they were asked to go to the homepage (task 5):

*Ah, I didn’t realize this was the homepage. It should be made clearer, perhaps with an overlay.*

[ID4, IC]

*A person who is usually tired, like a caregiver, should be able to use the program easily.*

[ID2, IC]

In addition, some participants did not understand the difference between a module and a chapter (task 6):

*I didn’t really understand the difference between “Module” and “Chapter.” The buttons to enter should be larger and clearer.*

[ID10, IC]

However, when participants landed on the selected chapter (task 6), first impressions were generally very positive, even though the quantity of text emerged as a potential issue:

*Wow, it seems like everything is there, though it’s a bit too much written.*

[ID7, IC]

Using shortcuts to return to previous chapters (task 10) or to move to others (task 7) was also perceived as complicated by some participants:

*It should be clearer how to go back.*

[ID5, FC]

*It should be more intuitive.*

[ID2, IC]

The exercises and activities were found easy to be carried out (task 9). While some participants appreciated the fact that automatic answers were provided, others would have appreciated more personalized feedback, especially for the open-ended questions:

*That’s fun! It makes me want to try all the answers too!*

[ID7, IC]

*It’s nice that no one is judging you, because it’s just you and your computer.*

[ID6, IC]

*Who is interested in what I’m writing? It would be nice if it could link to something else or to a specific answer.*

[ID1, IC]

### System Usability Scale

The average usability score using the SUS was 71.50 (SD 12.27), showing a good perception of iSupport Swiss usability. The most appreciated characteristic of the program was the perceived learnability of the website (mean score 4.00, SD 0.74; item 7: “I would imagine that most people would learn to use this system very quickly”), while the least appreciated was the perceived consistency and integration of functionalities (mean score 2.67, SD 0.07; item 6: “I thought there was too much inconsistency in this system”).

### Website Characteristics

Participants positively rated the website characteristics, with a mean score of 4.17 (SD 0.49) out of 5. The most appreciated features were related to the quality and quantity of information provided in the program with mean (SD) scores of 4.58 (0.51) and 4.42 (0.51), respectively. The least appreciated features were related to ease of navigation (mean score 3.42, SD 1.00) and ease of reading (mean score 4.08, SD 0.79). Table 3 summarizes scores from website characteristics and the SUS.

**Table 3.** Website characteristics and System Usability Scale (SUS) scores after using iSupport Swiss.

Characteristics	Value, mean (SD)	
SUS^a^	71.50 (12.27)	
Website characteristics^b^	31.17 (5.84)	
Quality of information	4.58 (0.51)	
Quantity of information	4.42 (0.51)	
Design and graphics	4.33 (0.65)	
Ease of reading	4.08 (0.79)	
Ease of navigation	3.42 (1.00)	

a Scores range from 0 to 100.

b Scores range from 1 to 5.

### List of Adjectives

Adjectives selected by participants to describe their first impressions about the program conveyed a general positive experience.

Out of a total of 50 impressions, the most selected attributes were “interesting” (75%) and “accessible” (50%), followed by “clear” (33%), “comprehensible” (33%), “engaging” (33%), and “structured” (33%). Only 1 participant selected negative adjectives such as “ambiguous,” “boring,” and “frustrating.”

### Open-Ended Questions

#### General Findings

Five main areas were identified through the analysis of postusability interviews: quality and relevance of the content, credibility of the source, navigation and usability, interactivity, and emotional impact. Table 4 summarizes the profiles of caregivers.

**Table 4.** Profile of caregivers by sex, age, type, and years of experience.

ID	Sex	Age (y)	Type of caregiver	Caregiver experience (y)
1	Female	58	Informal	6-10
2	Female	59	Informal	3-5
3	Female	75	Informal	6-10
4	Female	56	Informal	1-2
5	Male	32	Formal	6-10
6	Female	52	Informal	1-2
7	Male	57	Informal	1-2
8	Female	56	Informal	1-2
9	Male	55	Formal	>10
10	Male	59	Informal	6-10
11	Female	56	Informal	6-10
12	Female	63	Informal	1-2

#### Quality and Relevance of the Content

Both ICs and FCs particularly appreciated the purpose of the website, describing it as useful and innovative compared to existing resources.

All participants referred that they would use (or would have used) iSupport in the past, especially at the beginning of their caregiving journey:

*I would navigate it for a while…I would have used it at the initial stage; it would have been much more interesting because you are not prepared.*

[ID1, IC]

More specifically, the selection of topics and content was perceived as comprehensive, accessible, and closely aligned with their experiences, as highlighted by several participants:

*It is simple and reassuring because it provides advanced answers but presents them in a clear and accessible way, supporting those who want to learn more…we need this kind of program.*

[ID10, IC]

*I like the purpose…there is a need that may remain silent in family caregivers.*

[ID5, FC]

*There is a great selection, the chapters are not too overwhelming, and you have covered the essential points.*

[ID3, IC]

All participants would recommend iSupport Swiss to other caregivers, especially younger ones, noting that older caregivers might need assistance navigating the website:

*Certainly, I would recommend it to everyone, especially younger individuals, such as children, dealing with dementia, or even older ones, but with an introduction to guide them through.*

[ID8, IC]

#### Credibility of the Source

Additionally, 2 participants highlighted the credibility of the source as a key strength, noting that the fact that the program was created by the WHO and developed by the university contributed to a sense of trust and reassurance:

*Knowing that there is a credible website coming from the university to seek information is reassuring.*

[ID11, IC]

#### Navigation and Usability

Participants generally found the website functional and accessible; however, several usability issues emerged. Critical feedback mainly concerned navigation issues, as highlighted by participants during task performance. Some participants noted that it was not intuitive to switch from one module to another or to understand the differences between modules and chapters:

*Some navigation steps are a bit cumbersome…but, like many websites, with trial and error, you can find the information.*

[ID9, FC]

*Understanding how the chapters and subchapters work can be challenging; you can’t expect to arrive and immediately find an answer….*

[ID2, IC]

The same participant also suggested including a navigation guide to facilitate the navigation across the website:

*It’s a bit difficult to jump back and forth between the chapters; I would suggest a navigation guide.*

[ID2, IC]

#### Interactivity

The inclusion of closed-ended and open-ended questions was generally appreciated by most participants for the level of engagement and feedback regarding their roles as caregivers. For example, a user highlighted the potential of including case scenarios to rethink her own experiences:

*I really like the questions about situations that happen in daily life and receiving feedback…it’s important to reflect on oneself.*

[ID6, IC]

However, some participants expressed a preference for more personalized responses, particularly to open-ended questions:

*It would be helpful if someone provided a response in the exercises or if there were an option to share one’s own experience.*

[ID4, IC]

In addition, both FCs who took part in the study expressed their belief that they could assist and guide other caregivers at home in accessing the platform. However, they emphasized the need for additional time to do so effectively, as noted by a participant:

*I could provide more input on complex situations and support the caregiver to help them understand how it works, as a consultant…the problem is always time, which is barely enough to do what needs to be done.*

[ID9, FC]

#### Emotional Impact

In general, the overall user experience was positive: several users emphasized the potential of iSupport to provide relief and enhance self-efficacy as caregivers, while also facilitating a reframing of past experiences, as well as being documented by 3 participants:

*It was a bit like going back in time and, in the end, telling myself, “Many things could have been better, but it didn’t turn out that bad.”*

[ID12, IC]

*It made me feel good…based on the responses I received, I got confirmation that I’m on the right track, I give myself a pat on the back.*

[ID1, IC]

*It was relaxing…the images and feedback give you the feeling that there is always a solution.*

[ID6, IC]

### Integrated Synthesis of Quantitative and Qualitative Findings

The integration of quantitative and qualitative findings provides a comprehensive understanding of the usability of iSupport Swiss by linking observed task performance and usability scores with users’ lived experiences and perceptions.

Overall, quantitative task performance data indicated that most participants were able to complete the majority of tasks successfully or with minimal support, with success rates exceeding 70% for most activities. Qualitative data from think-aloud comments and interviews helped explain these results, revealing that participants generally perceived the platform as useful, credible, and reassuring, despite encountering specific navigation challenges.

In particular, tasks associated with lower completion rates or a higher need for assistance, such as changing language settings, navigating between modules and chapters, and using shortcuts, were reflected in qualitative feedback describing confusion related to information hierarchy, labeling, and navigation cues.

These qualitative insights contextualize the lower quantitative scores observed for ease of navigation and system consistency, highlighting concrete interface elements that limited smooth interaction with the program.

Conversely, high completion rates for tasks related to reading content and completing exercises were aligned with qualitative findings emphasizing the clarity, relevance, and emotional resonance of the content. Participants’ comments illustrated how interactive exercises and case-based examples supported reflection and self-efficacy, complementing quantitative indicators of good learnability and information quality.

This integrated analysis demonstrates how quantitative usability metrics identified where usability issues occurred, while qualitative findings explained why these issues emerged and how they affected users’ experiences.

## Discussion

### Principal Findings

This study aimed to assess the usability of iSupport Swiss (desktop version), a culturally adapted digital intervention designed to support ICs of people living with dementia. Overall, our findings indicate that iSupport Swiss is a user-friendly and valuable resource, with clear, credible content tailored to caregivers’ needs. Both FCs and ICs reported that the platform could complement existing support options, although several usability challenges, particularly related to navigation and personalization, were identified.

Usability scores indicated that the platform meets core usability criteria [27] and that participants found iSupport Swiss to be user-friendly and acceptable overall [27]. Moreover, task performance rates were above 70% for most tasks, suggesting an overall positive navigation experience. These results are consistent with findings on the usability of iSupport from Portugal and Brazil [23,31].

Nevertheless, several usability issues emerged during the task performance test, particularly regarding the ease of navigation and the content presentation. Specific tasks, such as modifying language settings, differentiating between modules and chapters, and using shortcuts to navigate back or forth, were associated with lower success rates or required support from the researchers. These difficulties were reflected in both behavioral observations and verbal feedback, pointing to challenges related to interface design and information hierarchy. At first glance, this may appear counterintuitive given that the digital literacy levels in our sample were relatively high, indicating that most participants possessed adequate skills to search, evaluate, and apply online health information. However, individual differences in eHealth literacy may help explain some of the usability challenges observed, particularly among those who required assistance during navigation tasks. Previous studies have similarly reported that even individuals with moderate-to-high digital literacy may encounter difficulties when engaging with novel or content-rich eHealth platforms, especially when the information structure or navigation paths are complex [22,32]. These findings suggest that future implementation strategies should consider providing introductory guidance or brief digital literacy reinforcement, particularly for older or less experienced caregivers, to ensure equitable access and sustained engagement with the program.

The think-aloud technique proved its anticipated complementarity with the other approaches we used. Caregivers commented freely on challenges encountered and offered suggestions to overcome navigation issues. Some of the most frequently reported issues were addressed following this study modality. For example, a welcome message was added to the login page to provide clearer orientation, and visual distinctions between modules and chapters were improved to enhance content clarity.

The posttest interviews complemented the qualitative findings by integrating qualitative insights into users’ experiences. More specifically, we collected diverging preferences regarding the interactive features of iSupport Swiss, highlighting the need for user-tailored integrations and personalization. Some participants appreciated the self-paced nature of the program and the lack of judgment or external pressure. Others expressed the desire for more personalized feedback and interaction with professionals, a finding that had already emerged during the cultural adaptation of iSupport Swiss [15].

Personalization is a well-discussed topic in the context of psychoeducational self-help programs. Some authors have argued that incorporating social components, such as human-based interaction, may improve the adherence of caregivers of people with dementia in using online interventions by mimicking analogical, humanized intervention-to-user interaction modalities [8].

However, iSupport was originally conceived by the WHO and was intended to function as a self-help tool that users can access at their own convenience and that does not require any additional support from external sources, under evidence-informed posits of greater flexibility of access and use, practicality, sustainability, and reduced stigma and inequality. Introducing personalized or human-mediated support would require substantial modifications to the program’s core structure and purpose, as well as significant investments in both technical and financial resources. Considering these constraints, with the goal of preserving the fidelity of the original program and in keeping with its assumed different potential reach through an eHealth platform, we decided to implement 2 measures. First, a disclaimer was added at the end of each exercise, clarifying that the automatic responses provided may not fully reflect the user’s individual situation. Second, updated and expanded links to external support resources were implemented, enabling users to seek more tailored assistance if needed.

Our study provides corroboration for the future implementation of iSupport. Participants highlighted the program’s clear and accessible language, comprehensive coverage of caregiver-relevant topics, and its overall credibility due to its institutional affiliations. These elements were perceived as reassuring and valuable, especially in contrast to existing resources for caregivers, which are often fragmented.

Importantly, the emotional dimension of usability also emerged as a key theme. Participants described feelings of reassurance, relief, and validation in their roles as caregivers when engaging with the content. The design and tone of the intervention appeared to foster a sense of self-efficacy and support, aligning with prior research emphasizing the role of affective responses in technology acceptance and sustained engagement [33].

While the digital format was generally well received, some participants emphasized the need for a multimodal approach to program dissemination, such as including a navigation guide or receiving support through health care professionals. In this regard, the engagement of health care professionals as facilitators of the program may represent a promising implementation strategy, though constraints related to time and workload must be addressed, as expressed by both FCs who took part in our study.

Finally, participants recommended iSupport Swiss, especially to caregivers who are caring for someone in the initial stage of dementia. This finding closely aligns with those described in the usability study of iSupport in Portugal [23] and during the adaptation work of iSupport Swiss [18].

This finding contrasts with existing literature showing that caregivers often tend to seek help only at later stages of the disease, as dementia progresses [34]. This discrepancy highlights an important opportunity: by promoting the dissemination of iSupport Swiss and encouraging its use earlier in the caregiving process, it may be possible to foster timely help-seeking behaviors and prevent the escalation of caregiver burden over time. Adjusting iSupport to the needs of caregivers at the early stages and facilitating access through the engagement of key stakeholders, such as family physicians and memory clinics, could therefore enhance its preventive impact and long-term effectiveness.

### Limitations

This study is not without limitations. First, the small sample size and the convenience sampling approach may limit the generalizability of the findings to the broader population of caregivers.

However, the sample size is consistent with those used in other usability studies of iSupport and eHealth interventions more broadly [13,23]. Moreover, the reduced number of participants allowed for an in-depth examination of each user’s interaction with the platform, enabling us to capture nuanced insights into the usability experience that might be overlooked in larger-scale evaluations.

Second, only 2 FCs were included in the study, which further limits the generalizability of the findings to this specific group. However, it is important to note that iSupport Swiss is primarily designed for ICs, who represent the main target audience of the intervention. In this sense, the limited number of FCs reflects the study’s focus. Nonetheless, their perspectives were particularly valuable, as they provided insights into how professional caregivers could play a supportive role in promoting, communicating, and integrating the use of iSupport among family caregivers.

Third, all participants were Italian-speaking and based in Ticino, a single region of Switzerland, which may not reflect the experiences of caregivers in other linguistic or cultural contexts. Nevertheless, it is important to note that this study was part of a broader project focused on the adaptation of iSupport to the Swiss context. As such, it serves as a foundational step to inform the development of a culturally relevant and linguistically accessible national intervention.

Fourth, regarding the measurement tools, it should be acknowledged that the use of self-reported questionnaires, such as the SUS and IT-eHEALS, may be subject to response bias or social desirability effects. Similarly, while the thematic analysis was conducted rigorously and independently by multiple researchers, qualitative interpretation inevitably involves some degree of subjectivity. Future work could combine these methods with more objective measures of user engagement, such as log-data analytics or longitudinal follow-ups, to further strengthen the validity of the findings.

Finally, the laboratory setting may not fully replicate the real-world conditions under which users engage with the platform, potentially influencing task performance and user feedback. At the same time, the controlled setting offered important methodological advantages: it enabled us to observe participants’ interactions in real time, ensure consistency across sessions, and promptly identify usability issues such as interface bugs or navigation errors. This allowed for a more accurate comprehension of the user experience and ensured that user difficulties were attributable to design features rather than external distractions or contextual variability.

### Conclusions and Future Directions

Our study contributes to and enriches the growing body of international research on iSupport, offering specific insights from the Swiss Italian-speaking context that complement findings from other implementation efforts in countries such as Spain [35], Portugal [23], Brazil [31], Australia [36], China [37], and the United Kingdom [38].

Our findings provide preliminary evidence supporting the usability and acceptability of iSupport Swiss among its target users. Several actionable insights were identified, particularly related to navigation, content clarity, and personalization. Future iterations of the platform should address these areas to ensure successful scaling and implementation. Additional research that involves more diverse samples, is conducted in naturalistic settings, and includes longitudinal assessments is needed to explore the sustained use and long-term impact of the intervention.

Beyond the iSupport framework, these findings have broader implications for the design, adaptation, and implementation of digital health interventions targeting caregivers and other vulnerable populations. They highlight the importance of combining rigorous usability testing with participatory and context-sensitive approaches to ensure that eHealth tools are not only technically functional but also culturally relevant, emotionally supportive, and accessible to users with diverse digital competencies. Our results suggest that successful digital interventions for caregivers must balance autonomy and guidance, offering self-paced learning while providing clear orientation and pathways for personalized support when needed. Moreover, the involvement of professional caregivers and health care providers as potential facilitators underscores the value of interdisciplinary collaboration in digital health innovation.

Ultimately, usability testing represents not only a methodological step in technology development but also a strategic investment in digital equity, ensuring that technological innovation translates into meaningful, inclusive, and sustainable support for caregivers and the communities they serve.

## Supplementary material

10.2196/81247 Multimedia Appendix 1 Screenshots from iSupport Swiss website version.

10.2196/81247 Multimedia Appendix 2 User guide during the usability session.

## Abbreviations

CAB: Community Advisory Board
FC: formal caregiver
IC: informal caregiver
IT-eHEALS: Italian version of the eHealth Literacy Scale
REDCap: Research Electronic Data Capture
SUS: System Usability Scale
WHO: World Health Organization
