# Supplementary material for: Usability of iSupport Swiss, a World Health Organization Digital Intervention for Caregivers of People With Dementia: Mixed Methods Study
Source: J Med Internet Res. 2026 Apr 15;28:e81247. doi: 10.2196/81247 (PMC13082442; doi:10.2196/81247)
Supplement: Multimedia Appendix 1 [file jmir-v28-e81247-s001.docx]

**Multimedia Appendix 1**


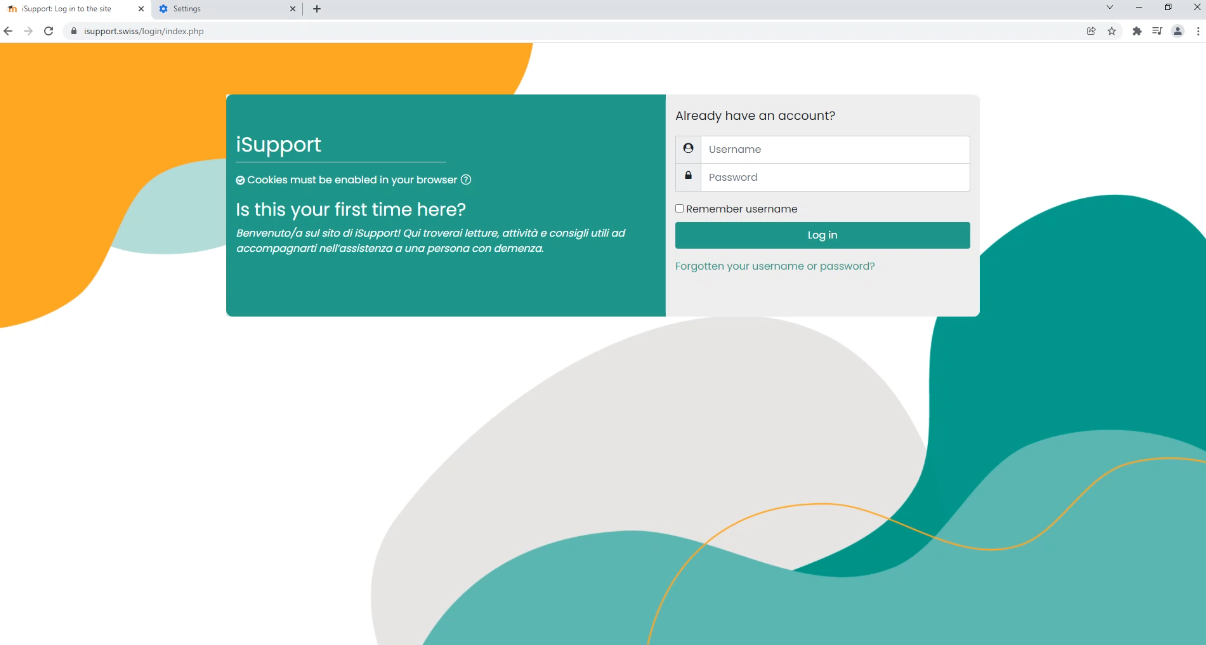


Figure S1. Login procedure


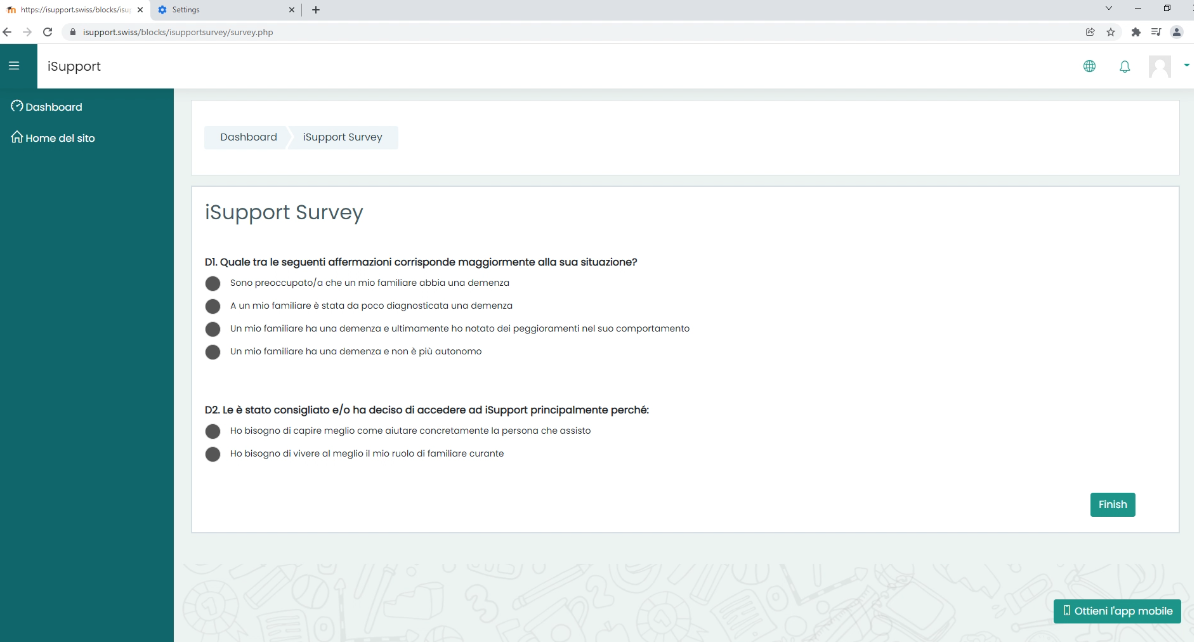
Figure S2. iSupport orientation survey


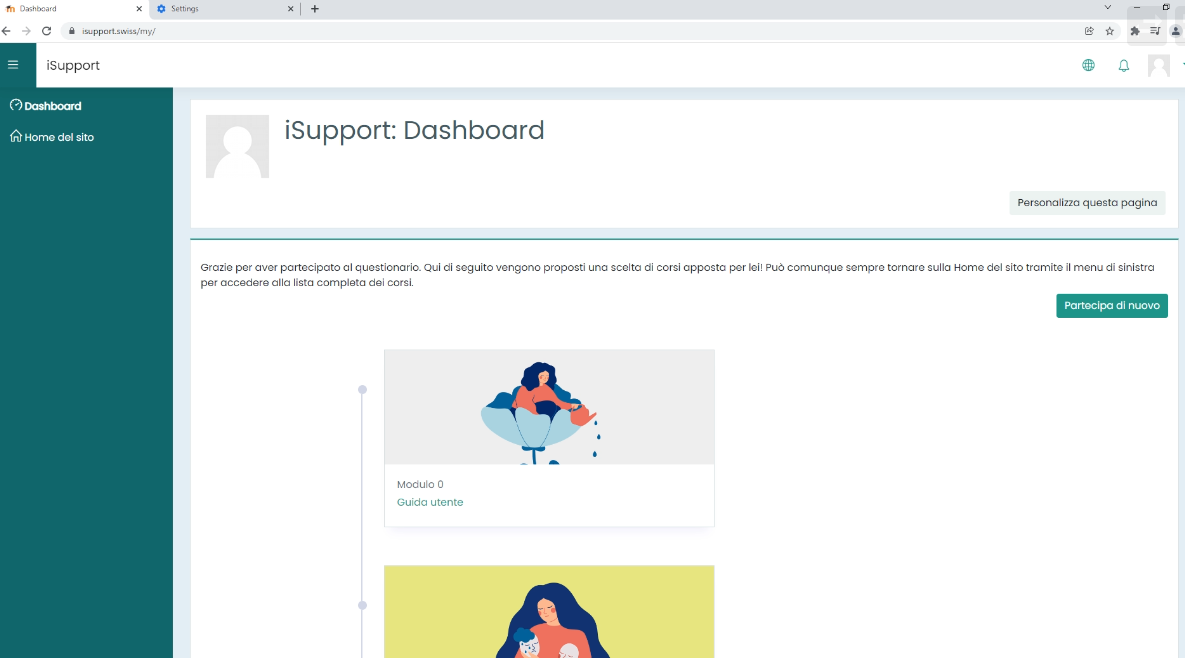


Figure S3. List of suggested courses


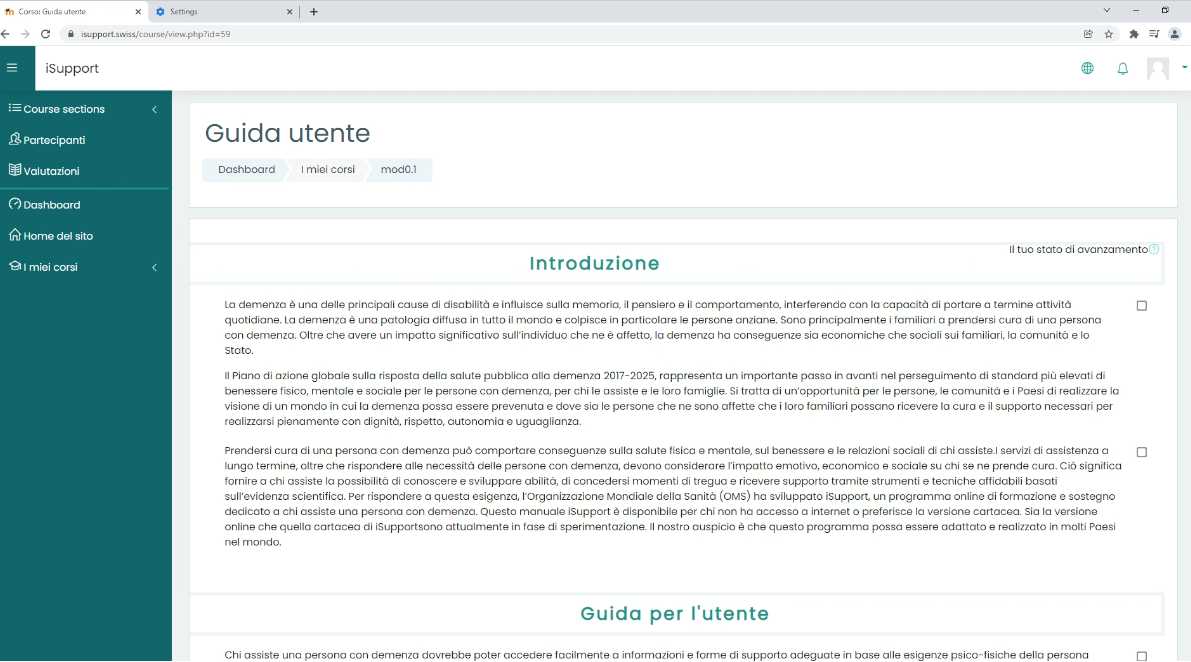


Figure S4. User guide


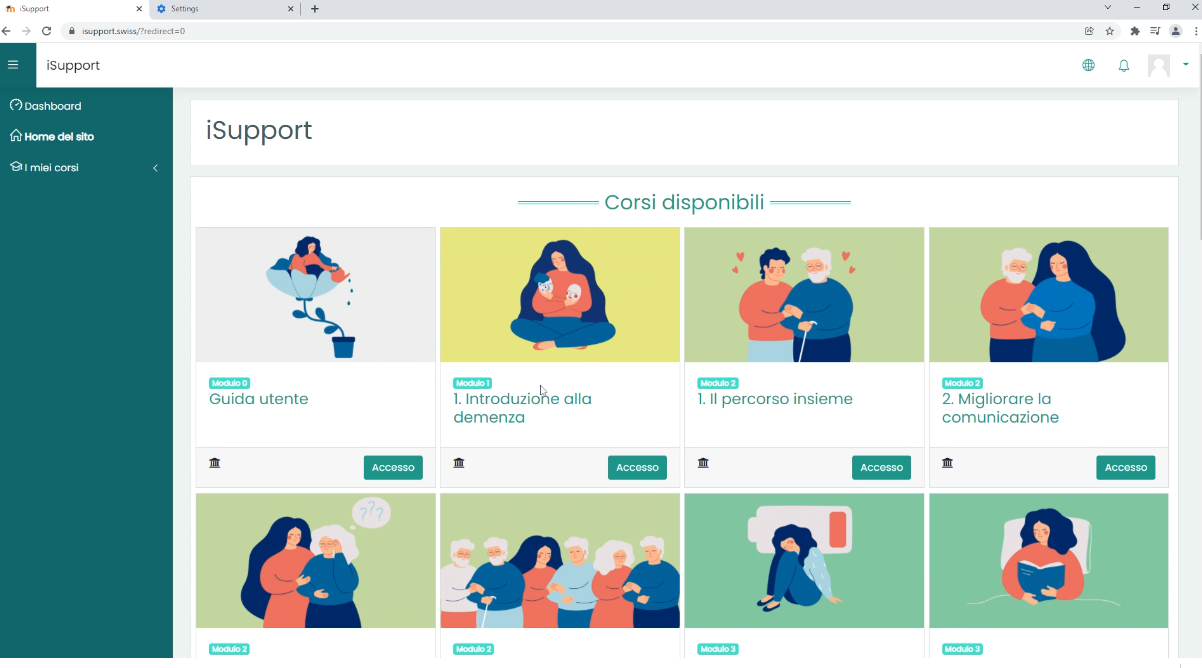


Figure S5. Homepage


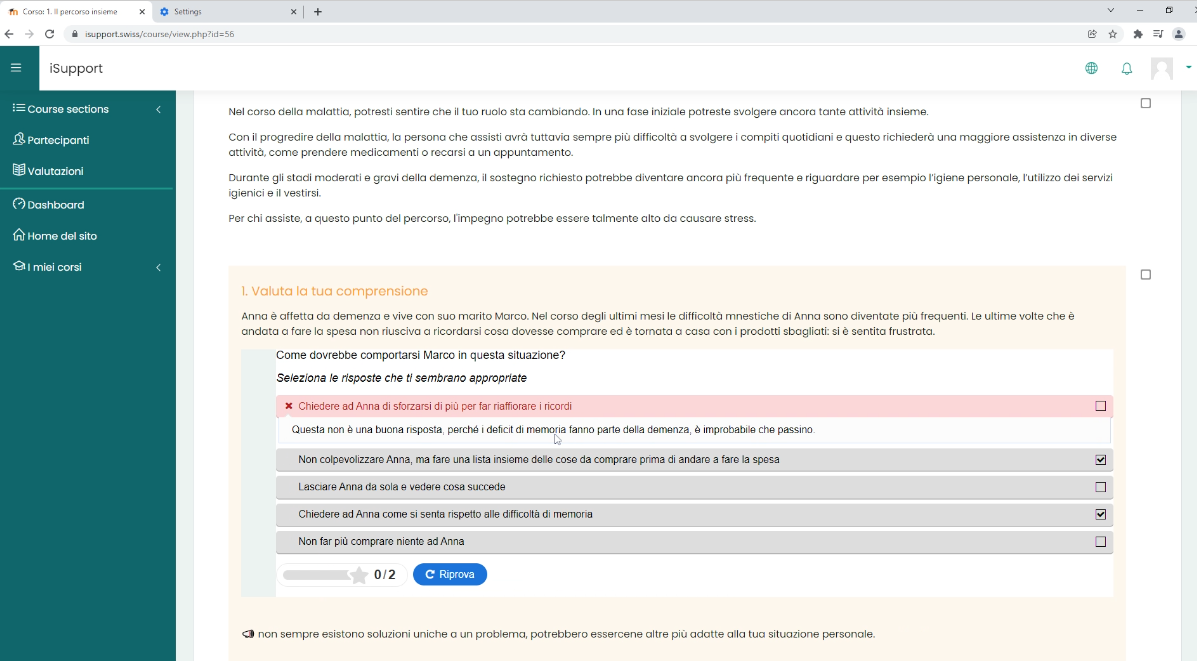


Figure S6. Multiple choice questions


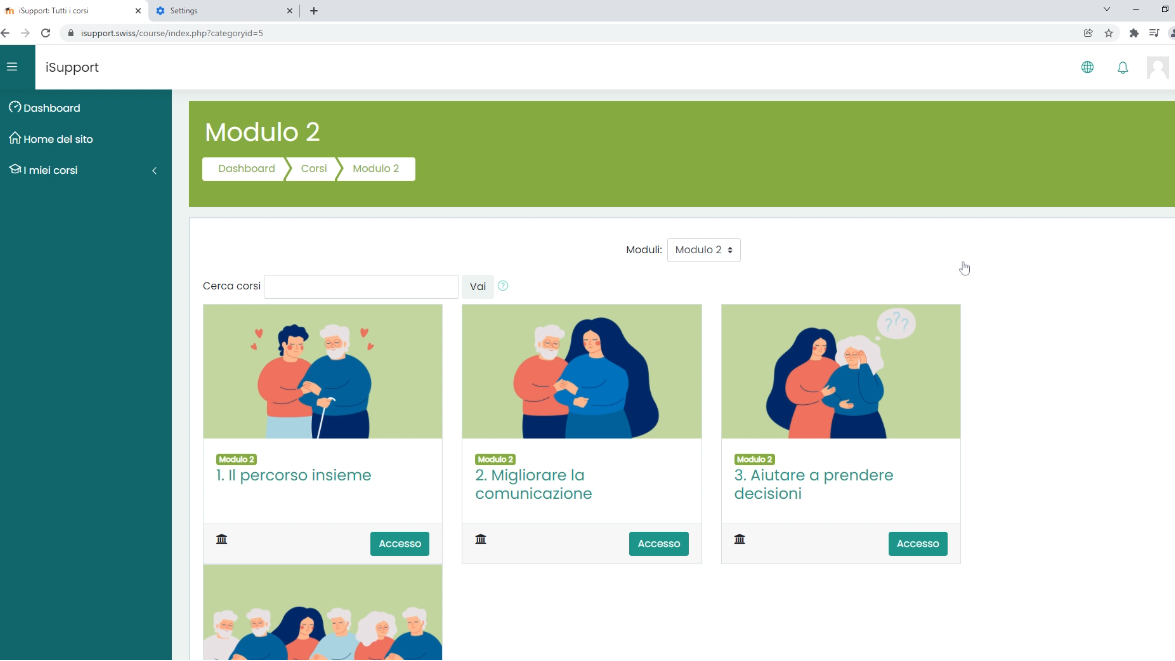


Open-ended questions

Figure S7. Module


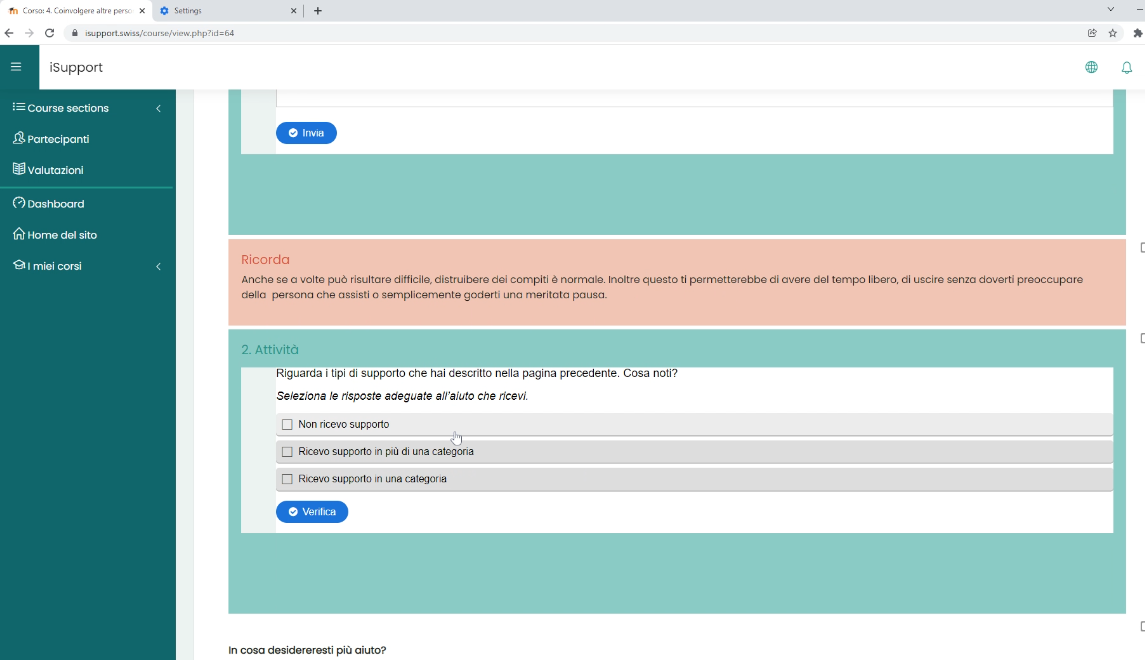


Figure S8. Open-ended questions


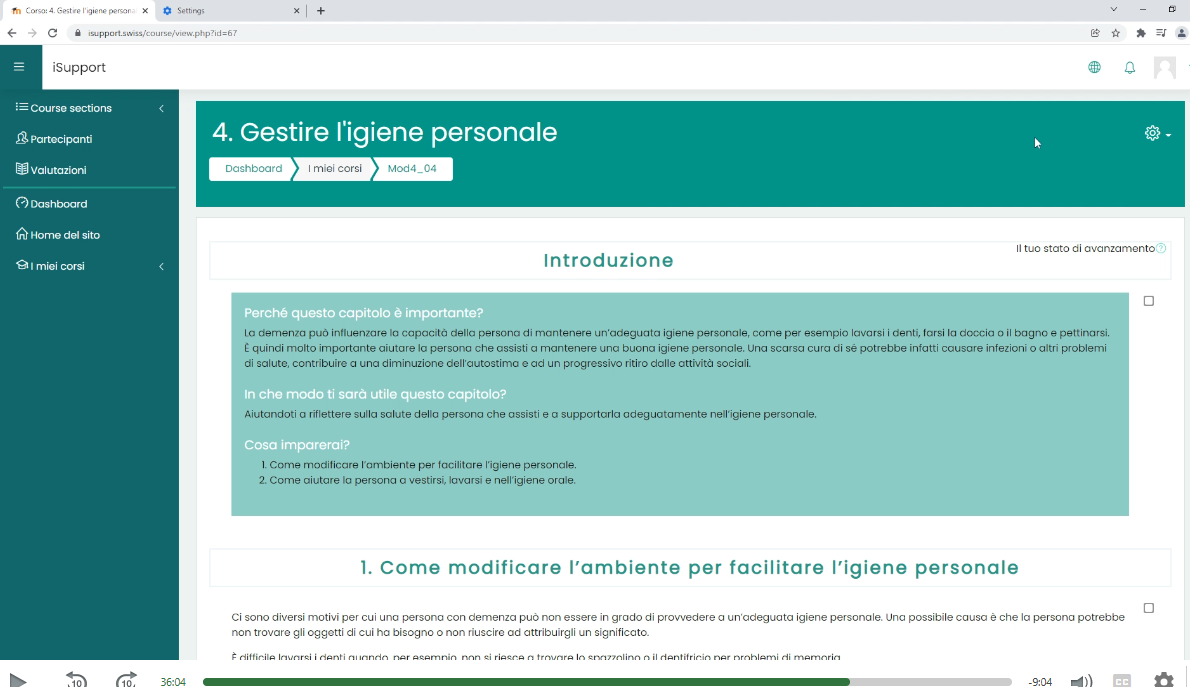


Figure S9. Chapter
