# Supplementary material for: Usability of iSupport Swiss, a World Health Organization Digital Intervention for Caregivers of People With Dementia: Mixed Methods Study
Source: J Med Internet Res. 2026 Apr 15;28:e81247. doi: 10.2196/81247 (PMC13082442; doi:10.2196/81247)

Università della Svizzera italiana

Lugano gen-feb 2022
Usability test
isupport@usi.ch

iSupport Prova di Usabilità

GUIDA PARTECIPANTE

SVOLGIMENTO

Durata: 90 minuti circa

- Introduzione al test
- Firma del consenso informato
- Compilazione domande pre sul sito RedCap
- Prova di iSupport
- Compilazione domande post sul sito Redcap
- Discussione

Prova di usabilità iSupport

1.

- Apra Internet e acceda al sito isupport.swiss
- Osservi la pagina per soli **5 secondi** e scriva i **primi tre aggettivi/frasi** brevi che meglio descrivono le sue impressioni:

2.

- Provi ora ad inserire le credenziali (username e password) e ad accedere al programma

*credenziali fornite da noi

3.

- Compili il questionario

4.

- Cosa farebbe ora?

- Si ritrova con i capitoli proposti? Sarebbe interessato a svolgerli?

5.

- Provi ad andare all’elenco generale dei moduli

6.

- Selezioni un modulo di interesse e poi un capitolo d’interesse

7.

- Apra il capitolo e se desidera lo scorra velocemente per vederne i contenuti

8.

- Cambi ora modulo e passi al capitolo e modulo da noi riferito

9.

- Legga il modulo e svolga la prima attività/esercizio proposto

10.

- Cosa farebbe per tornare al capitolo che ha già visionato?

11.

- Effettui il logout ed esca dal sito

GRAZIE PER AVER PARTECIPATO!

PUO’ RISPONDERE ALLE DOMANDE FINALI

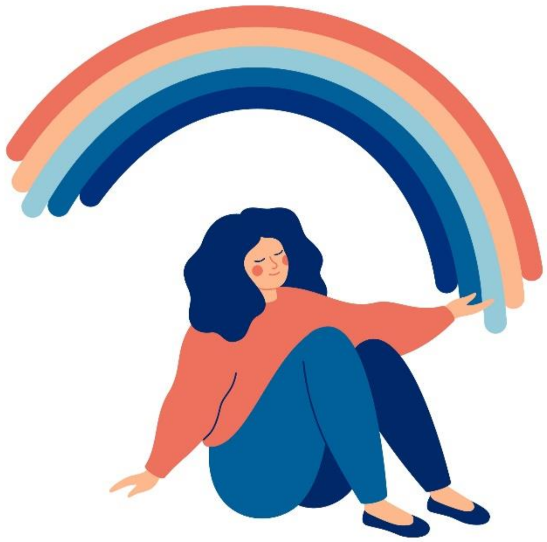

Supplement: Multimedia Appendix 2 [file jmir-v28-e81247-s002.pdf]
